# Effects on Blood Neurodegeneration Biomarkers of a Randomized Hearing Rehabilitation Intervention

**DOI:** 10.1093/geroni/igaf122.1081

**Published:** 2025-12-31

**Authors:** Nicholas Reed, James Pike, Jennifer Deal, Priya Palta, Josef Coresh

**Affiliations:** New York University Grossman School of Medicine, New York, New York, United States; New York University, New York, New York, United States; Johns Hopkins Bloomberg School of Public Health, Baltimore, Maryland, United States; UNC School of Medicine, North Carolina, United States; New York University, New York, New York, United States

## Abstract

The Aging and Cognitive Health Evaluation in Elders (ACHIEVE) study is a multicenter, parallel-arm, randomized trial (hearing intervention vs health education control) on 3-year cognitive decline among adults 70–84years with untreated hearing loss and without substantial cognitive impairment. Participants were recruited from: (1)a long-standing observational study (Atherosclerosis Risk in Communities [ARIC]), and (2)de novo community volunteers. In ACHIEVE, intervention slowed 3-year cognitive decline by 48% among ARIC participants only. To further investigate differences by treatment with respect to cognitive benefit, we tested the hypothesis that hearing intervention is associated with improved neurodegeneration blood biomarkers 3-years post-randomization. Plasma was collected in year 3 in a subsample (n = 540) while ARIC participants (n = 164) also had baseline plasma. Glial fibrillary acidic protein (GFAP) and neurofilament light (NfL) neurodegenerative biomarkers were derived using the Alamar CNS protein panel. Regression models estimated the association of treatment with 3-year biomarkers differences in the combined sample and by recruitment source and annualized change from baseline in the ARIC sample. Baseline characteristics were balanced by treatment. There were no significant differences on 3-year neurodegeneration biomarker levels by treatment in the combined or de novo groups. Among ARIC participants, intervention resulted in lower 3-year GFAP(mean:-0.415;95%CI:-0.712,-0.118) and NfL(mean:-0.349;95%CI:-0.652,-0.046). Annualized change from baseline analyses among ARIC participants revealed intervention was associated with a slower rate of increase in GFAP (difference-in-means: -0.060;95%CI:-0.112,-0.009). Hearing intervention was associated with positive 3-year effects on neurodegeneration blood biomarkers in ARIC participants which parallels cognitive decline results. Results provides objective evidence of brain changes following hearing intervention.

